# Endothelial-Mesenchymal Transition in Vascular Calcification of Ins2^Akita/+^ Mice

**DOI:** 10.1371/journal.pone.0167936

**Published:** 2016-12-09

**Authors:** Pierre J. Guihard, Jiayi Yao, Ana M. Blazquez-Medela, Luisa Iruela-Arispe, Kristina I. Boström, Yucheng Yao

**Affiliations:** 1 Division of Cardiology, David Geffen School of Medicine at UCLA, Los Angeles, California, United States of America; 2 The Molecular Biology Institute at UCLA, Los Angeles, California, United States of America; 3 Jonsson Comprehensive Cancer Center at UCLA, Los Angeles, California, United States of America; University of Illinois at Chicago, UNITED STATES

## Abstract

Endothelial-mesenchymal transition (EndMT) drives endothelium to contribute to normal development and disease processes. Here, we report that EndMTs occur in the diabetic endothelium of *Ins2*^*Akita/wt*^ mouse, and show that induction of sex determining region Y-box 2 (Sox2) is a mediator of excess BMP signaling that results in activation of EndMTs and increased vascular calcification. We also find an induction of a complex of serine proteases in the diabetic endothelium, required for the up-regulation of Sox2. Our results suggest that EndMTs contribute to vascular calcification in diabetic arteries.

## Introduction

Endothelial-mesenchymal transition (EndMT) is a process through which endothelial cells (ECs) transit into mesenchymal stem cells and gain plasticity for non-EC lineages [1, 2]. Previous studies have shown that EndMTs occur in organogenesis, such as neural crest formation and cardiogenesis [3, 4]. In disease, EndMTs have been demonstrated to contribute to the progress of pulmonary hypertension [5], atherosclerosis [6], cardiac and renal fibrosis [7, 8], fibrodysplasia ossificans progressive [9], and cancer progression [10]. Recently, our studies revealed a significant contribution of EndMTs in vascular calcification caused by deficiency of Matrix Gla protein (MGP), a well-established model of vascular calcification [2, 11, 12]. We showed that EndMTs drive endothelium to a mesenchymal state and directly contribute cells to the calcifying process in MGP-deficient aortas. These studies support EndMTs as a novel mechanism of EC contributing to vascular calcification [13].

Vascular calcification is a common complication of diabetes mellitus, and increases morbidity and mortality in diabetic patients [14]. Several lineages of vascular cells are known to contribute to diabetic calcification including smooth muscle cells and pericytes [15]. Recently, we demonstrated the presence of cells with EC-origin in calcified lesions in diabetic aortas by lineage tracing [14, 16], suggesting that diabetic endothelium not only produces osteoinductive factors, but directly contributes cells to the calcifying process. The process appears to be driven by bone morphogenetic proteins (BMPs) induced by high glucose in ECs [2, 11, 14], and is limited by enhanced BMP inhibition [16]. However, it is still unclear how the ECs gain plasticity to undergo osteogenesis in the setting of high glucose.

The *Ins2*^*Akita/wt*^ mouse is a monogenic diabetic model, and a model of diabetic calcific vasculopathy. The Akita mutation disrupts the two disulfide-bonds of A and B chains, which decreases proinsulin formation and in turn mature insulin [17]. *Ins2*^*Akita/wt*^ mice become spontaneously diabetic due to the reduced insulin level beginning at 3–4 weeks of age, and has been used as a model for type I diabetes mellitus (DM1) [18].

To determine if EndMTs play a role in calcific vasculopathy in diabetes mellitus, we investigated the emergence of EndMTs in aortic endothelium of *Ins2*^*Akita/wt*^ mice. We demonstrated that induction of sex determining region Y-box 2 (Sox2) mediated the increased expression of markers for EndMTs in the diabetic endothelium. Limiting endothelial Sox2 reduced the expression of these markers as well as aortic calcification. Our results also supported that a complex of serine proteases was involved in the induction of Sox2. Together, the results suggest that EndMTs contribute to vascular calcification in diabetes mellitus.

## Materials and Methods

### Animals

*Ins2*^*Akita/+*^ (C57BL/6-Ins2Akita/J), *Cdh5*^*Cre*^ (B6.Cg-Tg(Cdh5-cre)7Mlia/J) and *Sox2*^*flox/flox*^ (Sox2tm1.1Lan/J) mice were obtained from the Jackson Laboratory. Genotypes were confirmed by PCR [17, 19, 20], and experiments were performed with generations F4-F6. Littermates were used as wild type controls. All mice were fed a standard chow diet (Diet 8604, HarlanTeklad Laboratory). The studies were reviewed and approved by the Institutional Review Board and conducted in accordance with the animal care guideline set by the University of California, Los Angeles. All procedures were reviewed and approved by the Animal Care Committee (ARC) and the UCLA School of Medicine. The investigation conformed to the National Research Council, *Guide for the Care and Use of Laboratory Animals*, *Eighth Edition* (Washington, DC: The National Academies Press, 2011). Diisopropylfluorophosphate (DFP) (Sigma-Aldrich) and serpina1 (Origene) were injected via tail vein or retro-orbital injection (20–50 ng/g, daily) as in previous studies [21, 22]. Injections in *Ins2*^*Akita/+*^ mice started at 36 weeks of age, and continued for 4 weeks.

The animals were observed once daily during the weeks and once daily on weekends. The parameters that were assessed include the following: weight loss, breathing difficulties, edema, hunched posture, restlessness, vocalizing, impaired mobility, failure to groom and unkempt appearance. The weight was measured and recorded every three days. If the weight decreased by 5%, we measured the weight daily. If the weight decreased by 10%, we euthanized the mouse as per approved protocols. None of the mice died or became ill prior to the experimental endpoints in these studies; 5–30% isoflurane was used for euthanasia of all animals included in these studies.

### Tissue culture and CRISPR/Cas9 genomic editing

Human aortic endothelial cells (HAECs) were cultured as previously described [23]. For treatment of HAECs, BMP-4 (40 ng/ml, R&D system) and glucose (22 nmol/L, Sigma-Aldrich) were used as before [11]. For MGP-depletion using CRISPR/Cas9 genomic editing, HAECs were infected by lentiviral vectors, which containing gRNA for exon 1 of the *Mgp* gene and Cas9 (Sigma-Aldrich). The infected cells were selected by puromycin. The positive clones were collected and expanded after 14 days of selection. The depletion of MGP was confirmed by real-time PCR.

### RNA analysis

Real-time PCR analysis was performed as previously described [24]. Glyceraldehyde 3-phosphate dehydrogenase (GAPDH) was used as a control gene [24]. Primers and probes for mouse MGP, BMP-4, Sox2, Kruppel-like factor 4 (Klf4), snail family zinc finger 2 (Slug or Snail2), stem cell antigen 1 (Sca1), cluster of differentiation (CD)10, and c-kit (also referred to as CD117), were obtained from Applied Biosystems as part of Taqman^®^ Gene Expression Assays.

### Pre-sorting of ECs

The pre-sorting of aortic ECs was performed as previously described [11]. Briefly, the aortas were perfused with dispase and enzymatically dispersed. Then, the aortas were dissected into small pieces, and incubated for 45 minutes prior to fixation, staining and FACS analysis.

### Immunoblotting

Immunoblotting was performed as previously described [25]. Equal amounts of cellular protein or tissue lysates were used. Blots were incubated with specific antibodies to elastase 1, kallikrein 1 and 6 (all 200 ng/ml; Sigma-Aldrich), elastase 2 (200 ng/ml; Abgent), kallikrein 2 (300 ng/ml; Abgent), kallikrein 5 (300 ng/ml; Acris Antibodies), c-kit (200 ng/ml; Cell Signaling Technology), Sca1 (200 ng/ml; Merck Millipore), CD10 (1:100; ThermoFisher), CD44, CD90 (both 200 ng/ml; Abcam), CD71 (1:200; ThermoFisher), pSMAD1/5/8, Sox2, Klf4, Slug (all 400 ng/ml; Cell Signaling Technology) or total SMAD (400 ng/ml; Santa Cruz Biotechnology). ß-Actin (1:5000 dilution; Sigma-Aldrich) was used as loading control.

### Chromatin immunoprecipitation (ChIP) assay

For each ChIP assay, approximately 10^6^ cells were crosslinked in 1% formaldehyde for 10 minutes at room temperature. Glycine was added to a concentration of 0.125 M to quench the crosslinking, and the cells were rinsed with ice-cold PBS, resuspended in lysis buffers, and sonicated to shear the crosslinked DNA to fragments ranging from 200–500 bp. Sonication was performed on a Misonix 4000 Sonicator with the samples kept in an ice water bath; 1/10-1/20 of the sonicated lysate was saved for input DNA extraction. The lysate was incubated with 1 μg anti-Sox2 (STEMCELL Technologies) or normal IgG (Abcam) antibodies at 4°C overnight. After adding 40 μl protein G magnetic beads, the lysate was further incubated for 2–3 hours. The beads were washed with washing buffers repeatedly, after which elution buffer was added and the beads were incubated for 15 minutes at 65°C. Both the immunoprecipitated and input DNA samples were incubated overnight at 65°C for reversal of crosslinking. The DNA samples were then purified by sequential phenol: chloroform: isoamyl alcohol (Sigma). The final DNA products were ethanol precipitated and the pellets were air-dried and dissolved in 10 mM Tris-HCl. The real-time PCR was used to detected Sox2 binding sites around the promoters of target genes.

### Quantification of aortic calcium

Total aortic calcium was measured using a calcium assay kit (Bioassay) as previously described [26].

### Electron microscopy

Transmission electron microscopy (TEM) and scanning electron microscopy (SEM) were performed as described [11].

### Statistical analysis

Data were analyzed for statistical significance by ANOVA with post hoc Tukey’s analysis. The analyses were performed using GraphPad Instat^®^, version 3.0 (GraphPad Software). Data represent mean ± SD. P<0.05 was considered significant, and experiments were performed a minimum of three times.

## Results and Discussion

### EndMTs in aortic endothelium of *Ins2*^*Akita/+*^ mice

**Fig 1:**
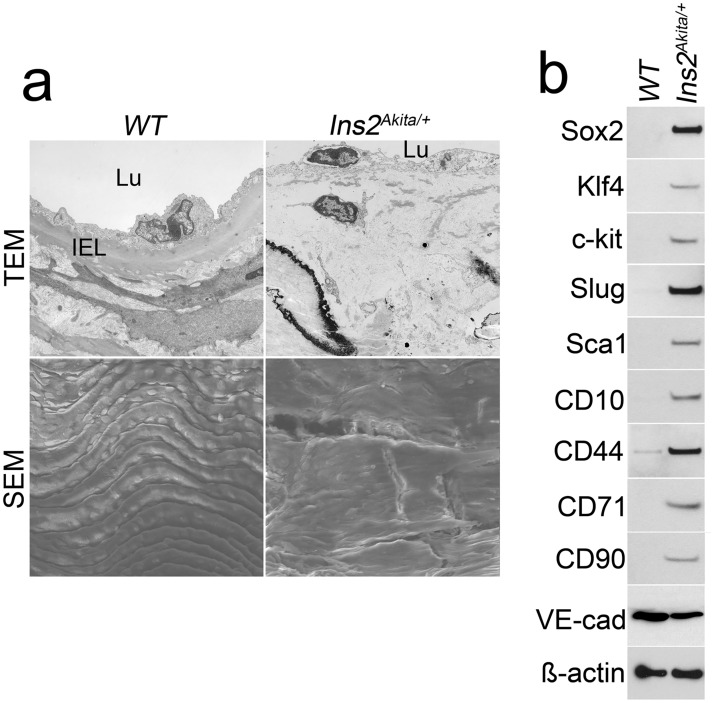
EndMTs in aortic tissue of *Ins2*^*Akita/+*^ mice. (a) Aortic endothelium in *Ins2*^*Akita/+*^ mice was examined by transmission electron microscopy (TEM) (top) and scanning electron microscopy (SEM) (bottom). Magnification for TEM, 3.7×10^3^. Magnification for SEM, 5×10^2^. Lu: lumen; IEL: Internal elastic lamina. (b) Expression of mesenchymal stem cell markers and EC marker VE-cadherin (VE-Cad) in CD31-positive and CD45-negative presorted cells from wild type (WT) and *Ins2*^*Akita/+*^ aortas at 40 weeks of age, as determined by immunoblotting.

We have previously shown that osteogenic cells of endothelial origin contribute to aortic calcification of *Ins2*^*Akita/+*^ mice [11]. To determine whether ECs undergo EndMTs as part of this process, we examined the aortic endothelium of *Ins2*^*Akita/+*^ mice at 40 weeks of age, when calcified lesions can be detected in the aortas [11, 14]. Using TEM and SEM, we observed that the internal elastic lamina (IEL), which is in close contact with endothelium, was degraded in the diabetic aortas (Fig 1a, top). The aortic endothelium was replaced by a mixture of cells, which appeared to be penetrating into the medial tissues. In addition, SEM showed what appeared to be ruptures in the diabetic endothelium (Fig 1a, bottom).

To examine whether EndMTs stimulated multipotent characteristics in the *Ins2*^*Akita/+*^ aortic endothelium, we analyzed the expression of stem cell and mesenchymal markers in isolated aortic ECs by immunoblotting. The EndMT-associated markers Sox2, Klf4, c-kit, Sca1, CD10, CD44, CD71, CD90 and Slug (also known as Snail2) were strongly induced in aortic ECs of *Ins2*^*Akita/+*^ mice (Fig 1b), while the expression of the EC marker VE-cadherin showed a decrease (Fig 1b). Thus, the results suggest that EndMTs cause a transition of the ECs to a multipotent state, which allows them to contribute cells to the calcifying process [11].

### Limiting Sox2 in ECs reduced EndMTs and vascular calcification in Ins2^Akita/+^ mice

**Fig 2:**
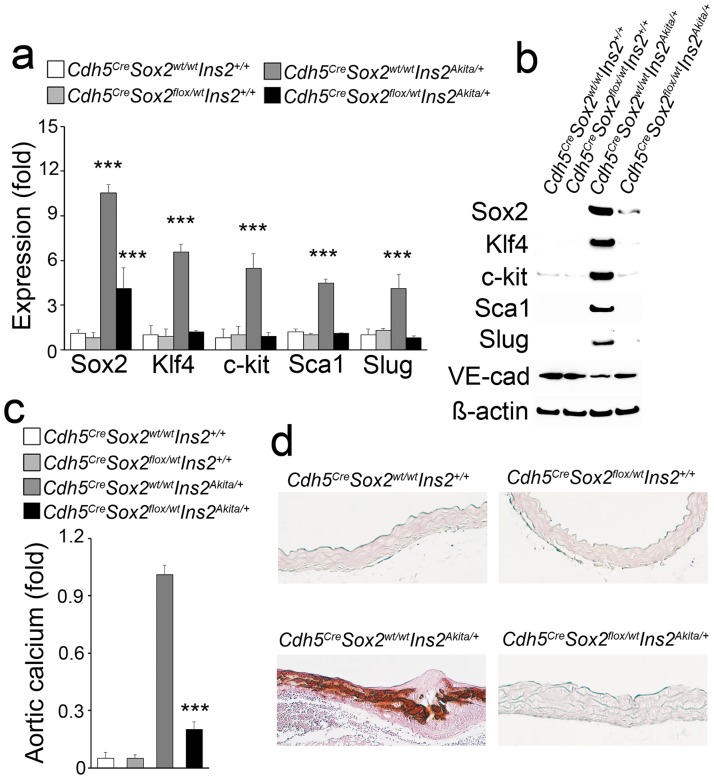
Limiting Sox2 in endothelium decreases EndMTs and calcification in *Ins2*^*Akita/+*^ aortas. (a-b) Decreased expression of Sox2, stem cell and mesenchymal markers in aortas of *Cdh5*^*Cre*^*Sox2*^*Flox/wt*^*Ins2*^*Akita/+*^, as shown by real-time PCR (a) and immunoblotting (b). VE-cad:VE-cadherin. (c-d) Total aortic calcium and aortic Alizarin red staining of *Cdh5*^*Cre*^*Sox2*^*wt/wt*^*Ins2*^*+/+*^ and *Cdh5*^*Cre*^*Sox2*^*Flox/wt*^*Ins2*^*+/+*^, *Cdh5*^*Cre*^*Sox2*^*wt/wt*^*Ins2*^*Akita/+*^ and *Cdh5*^*Cre*^*Sox2*^*Flox/wt*^*Ins2*^*Akita/+*^ mice. ***p<0.001.

Our previous studies demonstrated that Sox2 induction plays an essential role in EndMTs [2], and Sox2 expression is increased in the endothelium of *Ins2*^*Akita/+*^ mice (13). To determine whether reducing the levels of endothelial Sox2 would limit the induction of EndMT markers and calcification in *Ins2*^*Akita/+*^ mice, we bred *VE-cadherin* (*Cdh5)*^*Cre*^ and *Sox2*^*flox/flox*^ mice with *Ins2*^*Akita/+*^ mice. VE-cadherin-driven Cre expression was previously shown to reduce Sox2 in ECs in the *Sox2*^*flox/flox*^ mice [2]. We examined the aortic EC of *Cdh5*^*Cre*^*Sox2*^*flox/wt*^*Ins2*^*Akita/+*^ mice at 40 weeks of age, and confirmed that Sox2 expression was significantly decreased (Fig 2a and 2b). The endothelial expression of stem cell and mesenchymal markers was decreased as shown by real-time PCR and immunoblotting (Fig 2a and 2b). Furthermore, total aortic calcium and Alizarin red staining suggested significant improvement of aortic calcification in *Cdh5*^*Cre*^*Sox2*^*flox/wt*^*Ins2*^*Akita/+*^ mice. (Fig 2c and 2d). The results suggest that the Sox2 reduction limits EndMTs and vascular calcification in diabetic *Ins2*^*Akita/+*^ mice.

### Sox2 activates mesenchymal markers in HAECs with excess BMP activity

**Fig 3:**
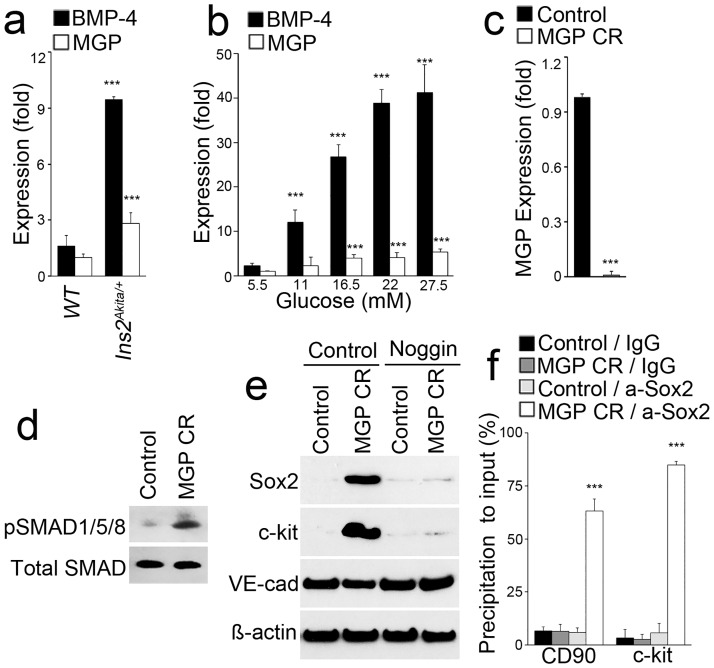
Sox2 activates CD90 and c-kit in MGP-deficient HAECs. (a-b) Expression of BMP-4 and MGP in endothelium of *Ins2*^*Akita/+*^ mice (a) and in HAEC (b) treated with different concentrations of glucose. (c-d) MGP expression and pSMAD1/5/8 level in HAECs after depletion of MGP by using CRISPR/Cas9 (MGP CR). (e) Expression of Sox2, c-kit and VE-cadherin (VE-cad) in MGP-depleted HAECs (MGP CR) with or without Noggin treatment. (f) ChIP assay shows abundant Sox2-binding in the promoters of CD90 and c-kit in MGP-depleted HAECs (MGP CR). a-Sox2: anti-Sox2 antibodies. ***, p<0.0001.

In previous studies, we demonstrated the induction of both BMP-4 and its inhibitor MGP in the endothelium of *Ins2*^*Akita/+*^ mice and glucose-treated human aortic endothelial cells (HAECs) [14]. We also found that excess BMP-4 as well as depletion of MGP triggered EndMTs [14]. However, when comparing the induction of BMP-4 and MGP in response to high glucose, we found a larger relative induction of BMP-4 compared to that of MGP in both the endothelium of *Ins2*^*Akita/+*^ mice and glucose-treated HAECs (Fig 3a and 3b). Even though these are relative induction levels, it suggests the MGP level is insufficient to counteract the BMP-4 in hyperglycemic conditions and might explain the Sox2 induction in diabetic conditions.

To confirm that MGP depletion affects Sox2 regulatory activity, as previously reported, we used CRISPR/Cas9 genomic editing system to deplete >99.5% of MGP RNA in HAECs. MGP depletion enhanced BMP signaling as determined by immunoblotting for pSMAD1/5/8 (Fig 3c and 3d). Sox2 and c-kit were induced in MGP-depleted HAECs, and Noggin abolished this induction (Fig 3e), suggesting that excess BMP signaling induce Sox2 to activate EndMTs. We subsequently performed ChIP assays with enrichment of the DNA by anti-Sox2 antibodies and examined the Sox2 binding sites in promoter regions of CD90 and c-kit by real-time PCR. As expected, the results showed abundant Sox2 binding around the regulatory regions of CD90 and c-kit in MGP-depleted HAECs as compared to controls (Fig 3f). Thus, a reduction of Sox2 would be expected to limit the reduction of EndMT markers.

### Up-regulation of serine proteases in aortic endothelium of *Ins2*^*Akita/+*^ mice

**Fig 4:**
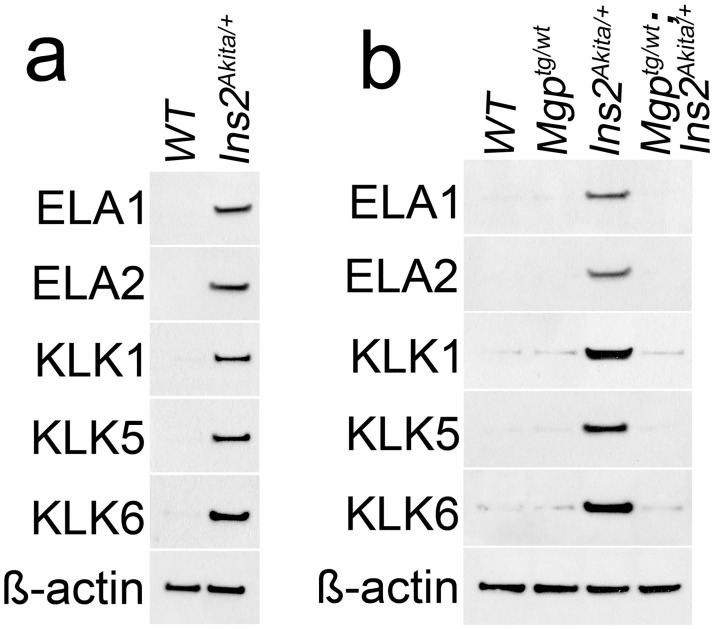
Induction of elastases and kallikreins in *Ins2*^*Akita/+*^ aortic endothelium. Expression of ELA 1, 2 and KLK 1, 5, 6 in CD31-positive and CD45-negative presorted cells (a) from wild type (WT) and *Ins2*^*Akita/+*^ aortas at 40 weeks of age, and (b) from aortas of WT, *Ins2*^*Akita/+*^, *Mgp*^*tg/wt*^ and *Mgp*^*tg/wt*^*Ins2*^*Akita/+*^ mice at 40 weeks of age, as determined by immunoblotting.

Our previous study demonstrated that a complex of serine proteases, which included elastase 1, 2 and kallikrein 1, 5 and 6, was involved in aortic calcification in MGP-deficient mice [2]. To determine if these proteases were involved in diabetic calcification, we examined expression in isolated aortic ECs from *Ins2*^*Akita/+*^ mice. The results revealed induction of the same five serine proteases also in diabetes, as determined by immunoblotting (Fig 4a).

To determine whether the induction of these serine proteases could be limited, we bred the *Ins2*^*Akita/+*^ mice with *Mgp*^*tg/wt*^ mice to suppress BMP activity [27], and examined the protease expression in isolated aortic ECs from *Mgp*^*tg/wt*^*Ins2*^*Akita/+*^ mice. The result showed a significant reduction in the expression of elastase 1, 2 and kallikrein 1, 5 and 6, as detected by immunoblotting (Fig 4b), suggesting that the induction involves by BMP activation.

### Inhibition of serine proteases decreased aortic calcification in *Ins2*^*Akita/+*^ mice

**Fig 5:**
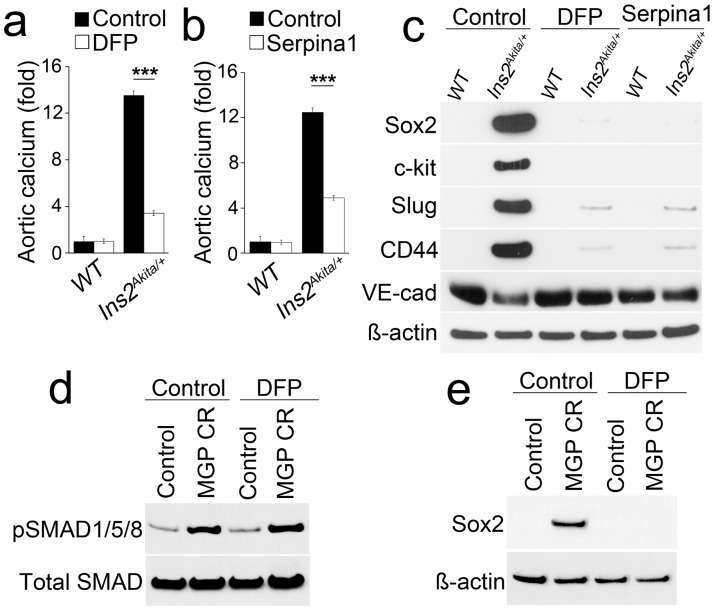
Inhibition of serine proteases decreases EndMTs and vascular calcification. (a-b) Total aortic calcium of *Ins2*^*Akita/+*^aortas after treatment of (a) diisopropylfluorophosphate (DFP) or (b) serpina1. ***p<0.001. (c) Expression of stem-cell markers in isolated aortic ECs of *Ins2*^*Akita/+*^ mice after treatment with diisopropylfluorophosphate (DFP) or serpina1, as determined by immunoblotting. (d-e) pSMAD1/5/8 levels and Sox2 expression in MGP-depleted HAECs after diisopropylfluorophosphate (DFP) treatment shown by immunoblotting.

To determine if we could limit calcification in the *Ins2*^*Akita/+*^ mice, we treated *Ins2*^*Akita/+*^ mice with the serine protease inhibitors DFP and serpina1 for 4 weeks starting at 36 weeks of age. We found that total aortic calcium was significantly reduced after DFP or serpina1 treatment (Fig 5a and 5b), suggesting that serine protease inhibitors are effective in limiting calcification. We also analyzed the expression of mesenchymal markers in aortic tissues by immunoblotting, and found significant decreases in Sox2, c-kit, Slug and CD44 in *Ins2*^*Akita/+*^ aortic ECs after protease inhibition (Fig 5c). We also found that DFP significantly decreased Sox2 induction in MGP-depleted HAECs, but have no effect on BMP activity (Fig 5d and 5e). Together, the results suggest that BMP-induced serine proteases are instrumental in the up-regulation of Sox2 and EndMTs in diabetes.

This study provides evidence that EndMTs contributes to vascular calcification in diabetic mice. Our data demonstrate degradation of IEL, loss of normal EC morphology in the aorta, and expression of mesenchymal stem cell markers. The EndMTs resemble those previously reported in *Mgp*^*-/-*^ mice, which exhibit extensive arterial calcification [2]. *Ins2*^*Akita/+*^ and *Mgp*^*-/-*^ mice share a similar phenotype of arterial medial calcification, which is in part promoted by excess BMP activity albeit through different mechanisms [11, 14]. In *Ins2*^*Akita/+*^ mice, excess vascular BMP activity results from induction of BMPs and BMP receptors by high glucose levels [14], whereas the excess BMP activity in *Mgp*^*-/-*^ mice is due to loss of BMP inhibition provided by MGP [28]. The excess BMP activity appears instrumental in inducing serine proteases that in turn activate Sox2 expression in ECs, and ultimately trigger EndMTs.

Recently, EndMTs have been associated with a number of diabetic complications. They have been shown to contribute to diabetic cardiomyopathy [29], diabetic nephropathy [30], diabetes-associated kidney fibrosis [31] and diabetic retinopathy [32] through various signal pathways. In the diabetic aorta, EndMTs are part of the calcific vasculopathy. We argue that excess vascular BMP activity contribute to at least two steps in the calcific process. BMP-4 is known to enhance stem cell characteristics in various stem cells [33] and activate the plasticity of ECs with the help of proteases and Sox2 [2]. Then, pro-osteogenic BMP2, which is induced in the diabetic media [14], may drive these cells into the osteogenic lineage with progressive calcification [34]. BMP regulation of proteases, Sox2 and EndMTs could also be associated with other diabetic complications, such as diabetic nephropathy, in which BMP5 has been associated with the activation of EndMTs [30] and polymorphism of Sox2 has significant gender-specific effects [35].

Activation of serine proteases, such as elastases and kallikreins, has been associated with vascular diseases. Elastases, a subgroup of serine proteases, break down elastin by cleaving peptide bonds at specific residues such as alanine, glycine, and valine [36]. Elevated activity of elastases promotes endothelial migration [37, 38], and is associated with the progression of pulmonary hypertension [39]. Elastase 2 is highly expressed in atherosclerotic lesions and participates in the degradation of elastin, fragments of which may enhance calcification [40–43]. Kallikreins, another subgroup of serine proteases, are classified according to biological function, as tissues kallikreins, which include kallikrein 1 to 15, and plasma kallikrein [44, 45]. Tissue kallikreins are expressed in endothelial cells and play roles in vascular formation and remodeling [46–48]. They are elevated in diabetes mellitus [49], and promote endothelial invasion in diabetic neovascularization [50–52]. Aortic levels were not documented in these studies. Our results support that complexes of elastases and kallikreins are intimately involved in the alteration of EC fate. However, the specific mechanism of these five proteases is not understood at this time, but may be related to activation of specific receptors or other key proteins in endothelial lineage differentiation.

## Conclusions

We show that BMP-induced serine proteases up-regulate Sox2, and Sox2-mediated EndMTs play a critical role in diabetic vascular calcification. Our results show the importance of EndMTs in diabetic vascular calcification, a common clinical problem, and provide information for developing new treatment strategies.
